# Long non-coding RNA Lnc-LALC facilitates colorectal cancer liver metastasis via epigenetically silencing LZTS1

**DOI:** 10.1038/s41419-021-03461-w

**Published:** 2021-02-26

**Authors:** Chuan Zhang, Lu Wang, Chi Jin, Jiahui Zhou, Chaofan Peng, Yong Wang, Ziwei Xu, Dongsheng Zhang, Yuanjian Huang, Yue Zhang, Dongjian Ji, Wen Peng, Kangpeng Jin, Junwei Tang, Yifei Feng, Yueming Sun

**Affiliations:** 1 The First School of Clinical Medicine, Nanjing Medical University, Nanjing, Jiangsu PR China; 2 Department of General Surgery, The First affiliated Hospital of Nanjing Medical University, Nanjing, Jiangsu PR China

**Keywords:** Cancer metabolism, Gastrointestinal cancer

## Abstract

Colorectal cancer (CRC) is one of the most common cancers around the world and endangers human health seriously. Liver metastasis is an important factor affecting the long-term prognosis of CRC and the specific mechanism of CRLM (colorectal cancer with liver metastasis) is not fully understood. *LZTS1* has been found dysregulated in many cancers, especially in CRC. Theories suggested that hypermethylation of the promoter regions of *LZTS1* was responsible for *LZTS1* abnormal expression in multiple malignant tumors. Although the role of *LZTS1* in CRC cell proliferation has been reported, its role in CRLM remains unclear. Numerous studies reported Long non-coding RNA (lncRNA) could regulate the gene expression level by regulating gene methylation status in many tumors. However, whether there were lncRNAs could change the methylation status of *LZTS1* or not in CRLM was unknown. In this study, we aimed to investigate whether there are lncRNAs can regulate the expression of *LZTS1* through affecting DNA methylation in CRLM. We found that upregulated *Lnc-LALC* in CRC was negatively correlated with *LZTS1* expression, and *Lnc-LALC* could regulate *LZTS1* expression in both mRNA and protein level in our study. Functionally, *Lnc-LALC* enhanced the CRC cells metastasis ability in vitro and vivo through inhibiting the expression of *LZTS1*. Furthermore, the precise mechanisms exploration showed that *lnc-LALC* could recruit DNA methyltransferases (DNMTs) to the *LZTS1* promoter by combining with Enhancer of zeste homolog 2(EZH2) and then altered the expression of *LZTS1* via DNMTs-mediated DNA methylation. Collectively, our data demonstrated the important role of *Lnc-LALC/ LZTS1* axis in CRLM development.

## Introduction

Colorectal cancer (CRC) is one of the most common cancers worldwide. According to the data published in the Journal of Cancer in 2018, the incidence and mortality of CRC both rank third among all malignant tumors, which seriously endangers human health^1^. Liver metastasis is an important factor affecting the long-term prognosis of CRC, which occurs in more than 50% of CRC patients^2^, and accounts for 40-50% of all CRC deaths^3^. Despite significant developments in the diagnostic techniques and therapeutic strategies, the clinical outcomes of CRC with liver metastasis (CRLM) patients remain unsatisfactory and the specific mechanism of CRLM is not fully clear. Therefore, it is important to explore the molecular mechanism and biological targets of CRLM in order to improve the early diagnosis rate of CRC.

Leucine zipper tumor-suppressor gene 1 (*LZTS1*) gene was firstly detected in esophageal cancer by microsatellite technology in 1999^4^. It is located at chromosome 8p22 and encodes a 67 kDa leucine zipper protein in the normal tissues while its expression was frequently downregulated or deficient in multiple cancers such as gastric cancer, lung cancer, and breast cancer^5–7^. Previous studies have determined that *LZTS1* could suppress cell proliferation and inhibit tumorigenicity by regulating CDC25C, cdk1, PS473 Akt, and pT308 Akt^8,9^. In CRC, *LZTS1* inhibits CRC cell growth through *AMT-mTOR* signal pathway by silencing p27Kip and overexpressing cyclin D1^10^. Additionally, there were theories suggest that hypermethylation of the promoter region of *LZTS1* could induce the deficient of *LZTS1* in cancer cells^11,12^. However, the molecular mechanisms of *LZTS1* and its methylation in CRLM remain largely unknown.

Long non-coding RNA (lncRNA) is a class of non-coding RNAs with a transcript length longer than 200 bp, which can regulate the function of target genes through epigenetics, transcriptional regulation, and post-transcriptional regulation in the human genome^13,14^. LncRNA plays a key role in the development of tumor cell proliferation, invasion, migration, cell cycle, and other functions. Among these roles, epigenetic changes caused by methylation is an important pathway to change the expression of target genes^15,16^. Yoon JH et al. reported that lncRNA *LUCAT1* in esophageal squamous cell carcinoma (ESCC) maintained the expression stability of DNMT1 by regulating methyltransferase to improve the level of DNA methylation, thereby inhibiting the expression of a series of tumor-suppressor genes and promoting the progression and metastasis of ESCC^17^. *Lnc00441* has been proved to reduce *RB1* expression and inhibit the apoptosis of HCC cell lines, which is significantly related to the changes in gene methylation caused by DNMT3A regulation^18^. In CRC, Merry CR confirmed that *TCONS_00023265* can promote the development of CRC by increasing s-adenosine methionine synthetase expression by reducing alanine butylthioether^19^. In this study, we aimed to investigate whether there are lncRNAs can regulate the expression of *LZTS1* through affecting DNA methylation in CRC. Through clustering analysis and multiple histological verification, we firstly found that *LINC00922* had a significantly linear correlation with *LZTS1*. Tao Liang et al.reported that *LINC00922* could accelerate the proliferation, migration, and invasion of lung cancer via the *miRNA-204/CXCR4* Axis^20^. Xin Yue et al. confirmed that *LINC00922* aggravates the malignant phenotype of breast cancer by regulating the *microRNA-424-5p/BDNF* Axis^21^. Additionally, we found that *LINC00922* was significantly correlated with liver metastasis and may participate in the expression regulating the process of *LZTS1* in CRLM in our study. On the basis of the specifically correlation between *LINC00922* and *LZTS1* founded in our research, we named *LINC00922* as *LZTS1 a*ssociated LncRNA in CRC(*Lnc-LALC*).We found that *Lnc-LALC* could enhance the CRC cells metastasis ability in *vitro* and *vivo* through inhibiting the expression of *LZTS1*. Furthermore, the precise mechanisms showed that *lnc-LALC* could recruit DNA methyltransferases (DNMTs) to the *LZTS1* promoter by combining with Enhancer of zeste homolog 2 (EZH2). EZH2 is an important member of polycomb repressive complex 2 (PRC2) which can recruit long no coding RNAs to their target gene promoter in many tumors. In our study, we found that *lncLALC*-EZH2 conjugate in the *LZTS1* promoter could regulate the methylation level via DNMTs and then altered the expression of *LZTS1*.EZH2 played a very important intermediary role in this mechanism regulation process. Collectively, our findings deeply explored the biologic information network regulating CRLM and provided a more theoretical basis of early prediction, accurate diagnosis, and targeted therapy for CRLM.

## Results

### *LZTS1* expression level was negatively correlated with CRC metastasis

**Fig. 1:**
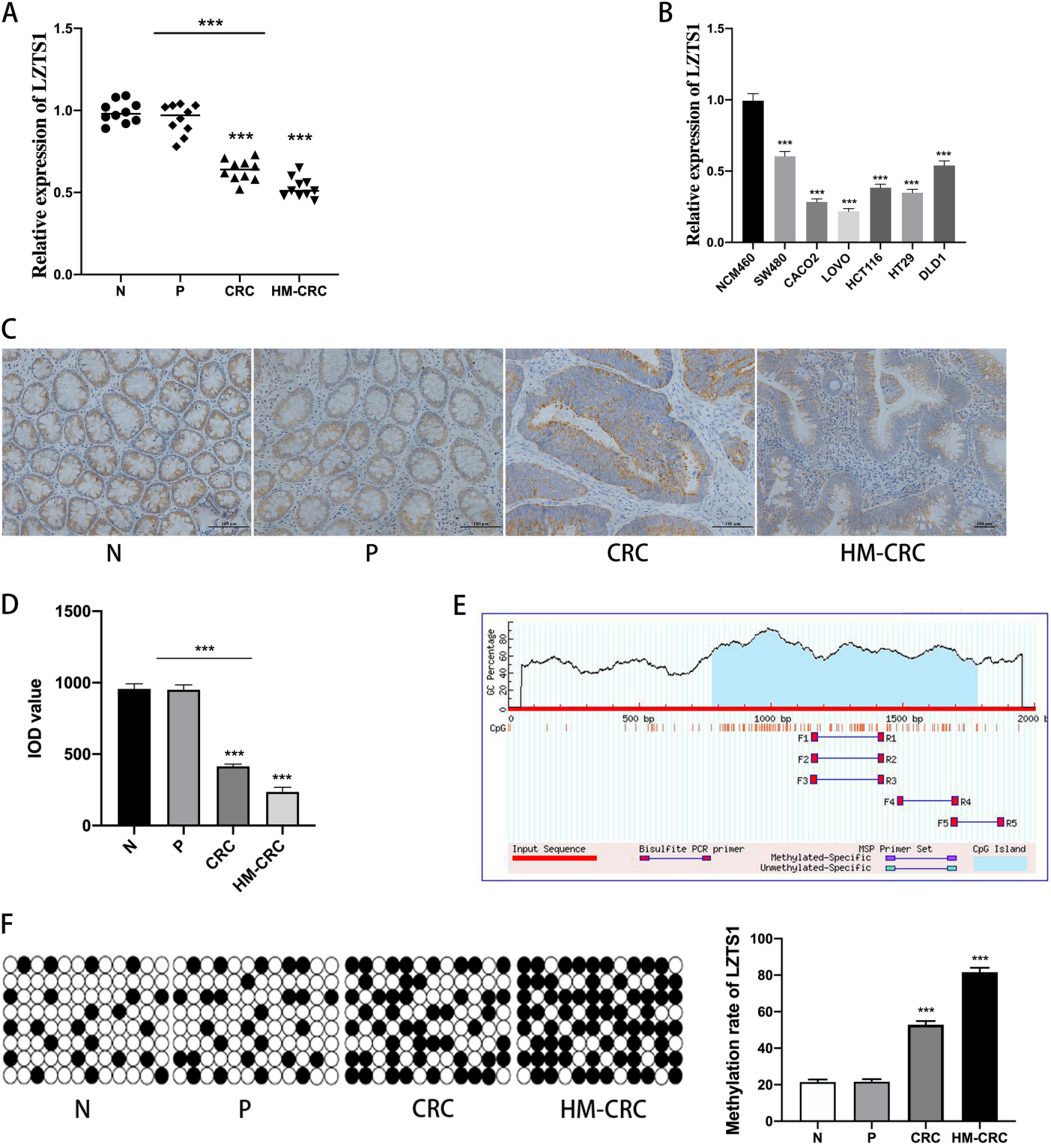
LZTS1 expression level was negatively correlated with CRC metastasis. **A** Relative expression of LZTS1 in the 40 tissues (normal intestinal epithelial tissues(N), non-metastatic paraneoplastic intestinal epithelial tissues(P), non-metastatic primary CRC tumor tissues(CRC) and primary tumor tissues with hepatic metastatic(HM-CRC),10 samples respectively) were assessed by qRT-PCR.. Statistical analysis was performed between group N and CRC,N and HM-CRC,N + P and CRC + HM-CRC groups. **B** The LZTS1 expression patterns in CRC cell lines and NCM460 cell lines were assessed by qRT-PCR. The LZTS1 expression level of NCM460 cells was used as a control. **C** Immunohistochemical assessment of LZTS1 expression in normal intestinal epithelial tissues(N), non-metastatic paraneoplastic intestinal epithelial tissues(P), non-metastatic primary tumor tissues(CRC) and primary tumor tissues with hepatic metastatic tissues(HM-CRC)(original magnification × 200). **D** Integrated optical density (IOD) value of the immunohistochemical assessment in samples. LZTS1 was down expressed in CRC samples and much more down expressed in HM-CRC samples compared with N samples. Statistical analysis was performed between group N and CRC, N and HM-CRC, N + P and CRC + HM-CRC groups. **E** The methylated CpG islands in the LZTS1 promoter region predicted by MethPrimer. **F** The methylation level of LZTS1 in four sample groups was detected by heavy sulfite sequencing and the methylation rate in the HM-CRC group was much higher than the other three groups. N group was used as a control. *P < 0.05; **P < 0.01; ***P < 0.001.

Firstly, we collected 10 samples of normal intestinal epithelial tissues(N), non-metastatic paraneoplastic intestinal epithelial tissues(P), non-metastatic primary tumor tissues (CRC), and primary tumor tissues with hepatic metastatic(HM-CRC), respectively. qRT-PCR and immunohistochemistry staining indicated that *LZTS1* expression level was negatively correlated with CRC metastasis (Fig. 1A, C, D). QRT-PCR results revealed that *LZTS1* was downregulated in CRC cell lines when the NCM460 cells were used as a control (Fig. 1B). Based on the preliminary research, we predicted the methylated CpG islands in the *LZTS1* promoter region by MethPrimer (http://www.urogene.org/methprimer/) and found that there were three important methylated CpG islands in the promoter region (Fig. 1E). Bisulfite sequencing indicated that the methylation level in the group with liver metastasis was much higher than the other three groups (Fig. 1F). It suggested that there may be some specific mechanisms to promote the methylation of the *LZTS1* promoter region in the progression of CRC metastasis which further causing *LZTS1* inhibition and turning off its anticancer effects.

### *Lnc-LALC* was upregulated in CRC cells and positively correlated with metastatic factors

**Fig. 2:**
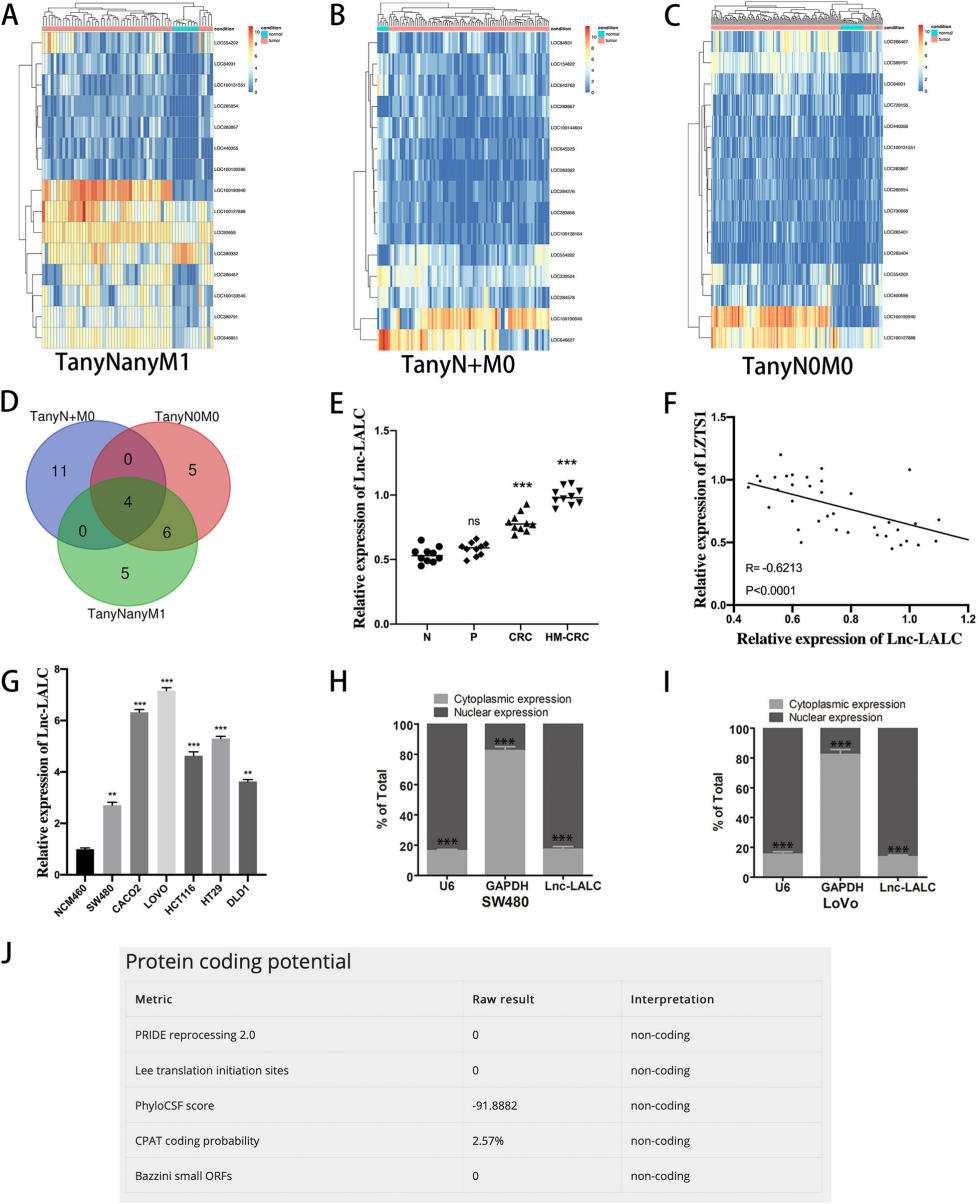
Lnc-LALC was upregulated in CRC cells and positively correlated with metastatic factors. **A** The top 15 dysregulated lncRNAs in TanyNanyM1 group were shown by hierarchical clustering analysis. **B** The top 15 dysregulated lncRNAs in TanyN+M0 group were shown by hierarchical clustering analysis. **C** The top 15 dysregulated lncRNAs in TanyN0M0 group were shown by hierarchical clustering analysis. **D** VENNY analysis identified 4 lncRNAs which were all aberrantly regulated in the three comparisons (TanyNanyM1, TanyN+M0, and TanyN0M0). **E** Relative expression of LINC00922 in the above 40 tissues was assessed by qRT-PCR. N group was used as a control. **F** A negative correlation between expression levels of Lnc-LALC and LZTS1 in tissues was determined by Person analysis (*n* = 40, *r* = −0.6213, *P* < 0.0001). *** *P* < 0.001; **P < 0.01 and *P < 0.05. **G** The LINC00922 expression patterns in CRC cell lines and NCM460 cell lines were assessed by qRT-PCR. **H** Cellular characterization of lnc-LALC in SW480 cells, the levels of nuclear control transcript (U6), cytoplasmic control transcript (glyceraldehyde-3-phosphate dehydrogenase [GAPDH] mRNA), and lnc-LALC were assessed by qRT-PCR in nuclear and cytoplasmic fractions. Data are presented as a percentage of U6, GAPDH, and lnc-LALC levels and total levels for each were taken as 100%. **I** Cellular characterization of lnc-LALC in LoVo cells. **J** The non-coding property of Lnc-LALC was confirmed using bioinformatics software (http://cpc.cbi.pku.edu.cn/programs/run_cpc.jsp).

CRC microarray data of lncRNAs of 435 patients were downloaded from The Cancer Genome Atlas (TCGA). To obtain the differentially expressed lncRNAs in T_any_N_any_M1, T_any_N_+_M0, and T_any_N0M0 groups, we analyzed the TCGA datasets based on the corresponding clinical data. We identified 30 aberrant expressed lncRNAs in the T_any_N_any_M1 group, 17 aberrant expressed lncRNAs in the T_any_N_+_M0 group and 96 aberrant expressed lncRNAs in the T_any_N0M0 group (*p* < 0.05, FC > 2). The top 15 dysregulated lncRNAs in these 3 groups were shown by hierarchical clustering analysis in Fig. 2A–C. The overlapping results among the three groups identified 4 aberrant lncRNAs (Fig. 2D) (Supplementary Table 1). Next, we detected the expression of the 4 lncRNAs in the 40 samples by qRT-PCR assay and found that only *LINC00922* was positively correlated with CRLM and negatively correlated with *LZTS1* expression (*r* = −0.6213, *P* < 0.0001)(Fig. 2E, F); The expression pattern in CRC cell lines were also assessed by qRT-PCR and the results showed that *LINC00922* was upregulated in CRC cells. Among CRC cells, *LINC00922* was highest expressed in LoVo cells while lowest expressed in SW480 cells relatively (Fig. 2G).

Thus, LoVo and SW480 cells were used for future study. And then we selected *LINC00922* for further research and named it *Lnc-LALC* (LZTS1 associated LncRNA in CRC). Sublocation analysis indicated that *Lnc-LALC* was confined in nucleus in SW480 and LoVo cells(Fig. 2H, I). The non-coding property of *Lnc-LALC* was confirmed using bioinformatics software (Fig. 2J).

The relationship between *Lnc-LALC* expression and the clinicopathologic features of CRC were also analyzed. We divided 120 patients into two groups according to their average *Lnc-LALC* expression level in tumor tissues: low level (*n* = 60) and high level (*n* = 60). And we found that *Lnc-LALC* expression was positively correlated with nodal metastasis(*P* < 0.01, vascular invasion (*P* < 0.05), advanced stage(*P* < 0.01), liver metastasis (*P* < 0.01) and T stage (*P* < 0.01). In addition, *Lnc-LALC* expression was also correlated with the CEA level (*P* < 0.01). But we did not find statistical differences between *Lnc-LALC* expression and age, gender, or tumor size. These results indicated that *Lnc-LALC* was significantly positively correlated with the metastasis factors(Supplementary Table 2).

### *Lnc-LALC* promoted CRC cells migration and invasion in *vitro*

**Fig. 3:**
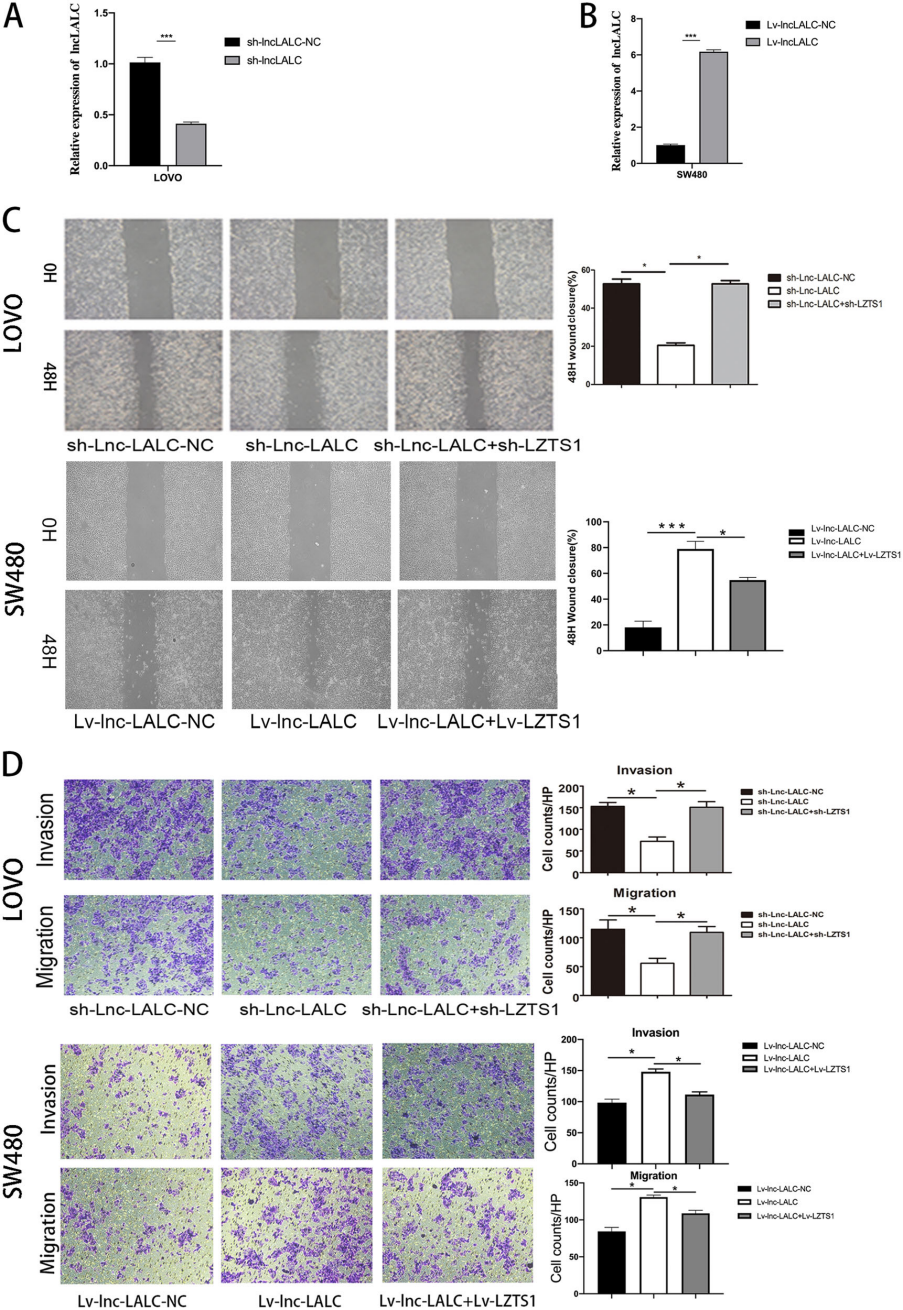
Lnc-LALC promoted CRC cells migration and invasion in vitro. **A** The knockdown efficiency of lncLALC by sh-lncLALC was assessed by qRT-PCR in LoVo cells. **B** The overexpression efficiency of lncLALC by Lv-lncLALC was assessed by qRT-PCR in SW480 cells. **C** Wound-healing assay showed that knockdown of Lnc-LALC inhibited the migratory ability of LoVo cells and knockdown of LZTS1 could recover this inhibiting effect; overexpression of Lnc-LALC promoted the migratory ability of SW480 cells and overexpression of LZTS1 could recover this promoting effect. *P < 0.05(original magnification × 40). **D** Transwell assay showed that knockdown of Lnc-LALC inhibited the invasive ability of LoVo cells and knockdown of LZTS1 could recover this inhibiting effect; overexpression of Lnc-LALC promoted the invasive ability of SW480 cells and overexpression of LZTS1 could recover this promoting effect(original magnification × 100). *P < 0.05. The data represent the mean ± SD from three independent experiments.

Firstly, we stably transfected LoVo and SW480 cells with different lentivirus vectors. qRT-PCR indated that sh-*lncLALC* could reduce *lnc-LALC* expression by 60% compared with sh-*lncLALC*-NC group and Lv-*lncLALC* could enhance *lnc-LALC* expression 6 times compared with Lv-*lncLALC*-NC group (Fig. 3A, B). The results of Wound-Healing and Transwell showed that the overexpression of *Lnc-LALC* could significantly promote the metastasis and invasion of SW480 cells and Lv-*LZTS1* could recover this promoting effect by overexpressing *LZTS1*. The metastasis and invasion of LoVo cells decreased significantly after *Lnc-LALC* shRNA treatment and the recovery experiments confirmed that silencing *LZTS1* expression by sh-*lncLALC* could restore the inhibition effects on metastasis and invasion caused by sh-*lncLALC* (Fig. 3C, D). This indicated that *Lnc-LALC* could regulate the ability of metastasis and invasion in CRC cells by regulating *LZTS1*.

### *Lnc-LALC* enhanced CRC cells metastasis ability in vivo

**Fig. 4:**
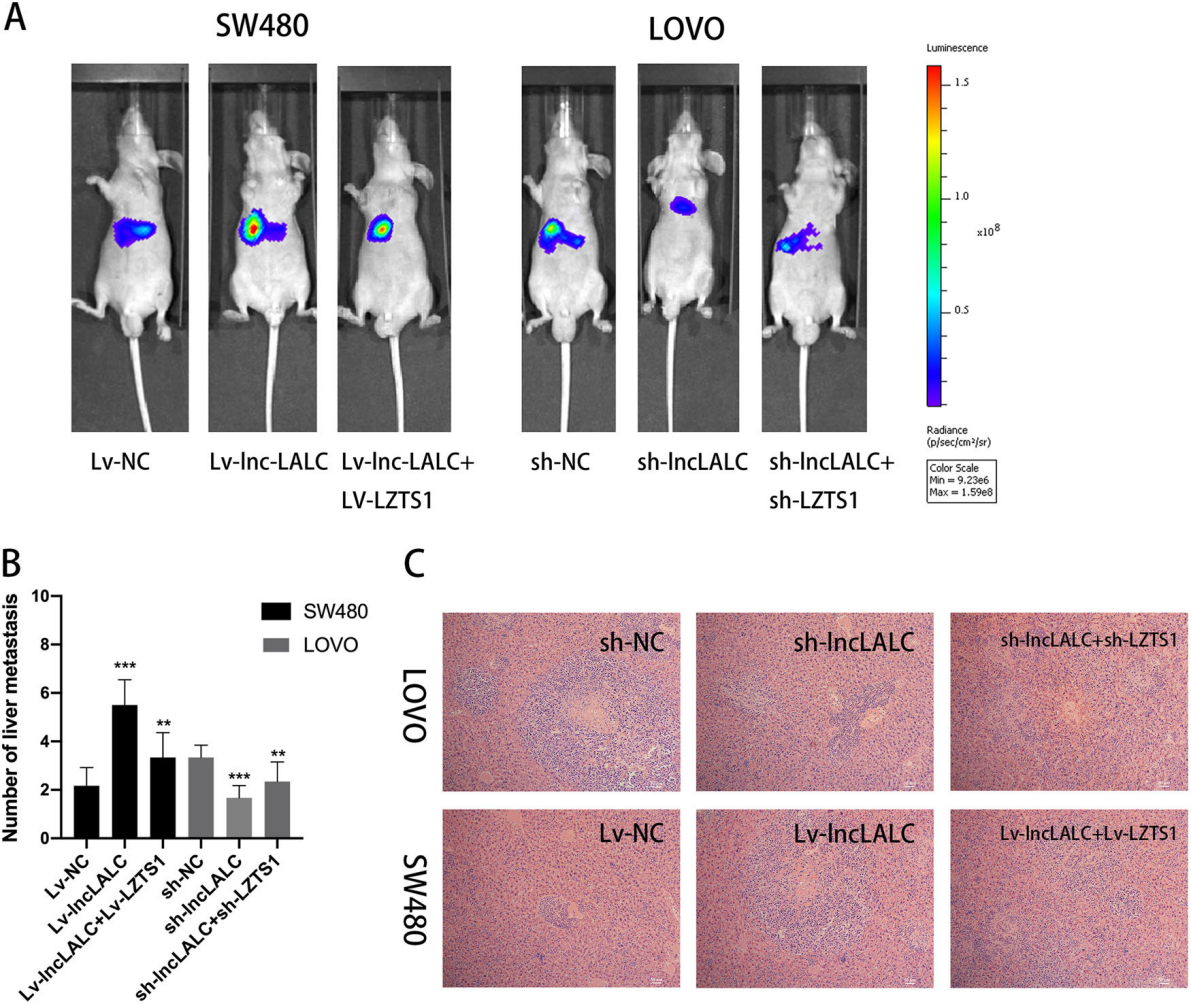
Lnc-LALC enhanced CRC cells metastasis ability in vivo. **A** Hepatic metastasis models were conducted by injecting CRC cells (2 × 10^7^ cells suspended in 200 μL PBS) into the spleen of 48 BALB/c nude mice in 6 groups (8 in each group) and liver metastasis was investigated respectively using the IVIS Lumina II system. Representative images in each group were presented. The fluorescence intensity was highest in Lv-lnc-LALC-SW480 group and lowest in sh-lncLALC-LoVo group. **B** Hepatic metastatic tumors in different groups with volumes >2 mm^3^ were identified and compared.Lv-NC-SW480 group and sh-NC-LoVo group were used as a control in two cell lines respectively. *P < 0.05; **P < 0.01; ***P < 0.001. **C** H&E staining confirmed the pathological pattern of the hepatic metastatic nodules in these six groups was metastatic adenocarcinoma(original magnification × 100).

To explore the function of *Lnc-LALC* in vivo, we conducted hepatic metastasis models according to the reported theories^22–24^. As shown in Fig. 4A, the fluorescence intensity was highest in Lv-*lncLALC*-SW480 group and lowest in sh-*lncLALC*-LoVo group. In Fig. 4B, we found that the number of liver metastatic nodules in the sh-*lncLALC*-LoVo group was significantly less than that of the sh-NC-LoVo group. The recovery experiments confirmed that silencing *LZTS1* expression could restore the inhibition effects caused by sh-*lncLALC*. In contrast, *lnc-LALC* overexpression promoted SW480 metastasis ability dramatically and the number of liver metastatic nodules in the Lv-*lncLALC*-SW480 group was much more than that of the Lv-NC-SW480 group. Additionally, Lv-LZTS1 could recover this promoting effect via overexpressing *LZTS1*. By H&E staining, we confirmed that the pathological pattern of the liver metastatic nodules in these six groups was metastatic adenocarcinoma (Fig. 4C). These results demonstrated that *Lnc-LALC* could enhance the CRC cells metastasis ability in vivo.

### *Lnc-LALC* regulated methylation of *LZTS1 promoters mediated by* DNA methyltransferases

DNA methylation is one important regulatory mechanism in gene epigenetics and plays an important role in gene transcription^25,26^. Studies have confirmed the presence of tumor-suppressor promoter methylation modification in various malignant tumors such as breast cancer, esophageal cancer, and CRC^27,28^. In addition, lncRNAs play key roles in various processes of tumor development and regulating the expression of target genes via methylation and then giving rise to epigenetic changes is an important way^15,16^. Our experimental results suggested that there was a significant correlation between *lnc-LALC* and *LZTS1* expression. So, we hypothesized *LZTS1* methylation may play important roles in CRC metastasis and *lnc-LALC* may participate in this progress.

**Fig. 5:**
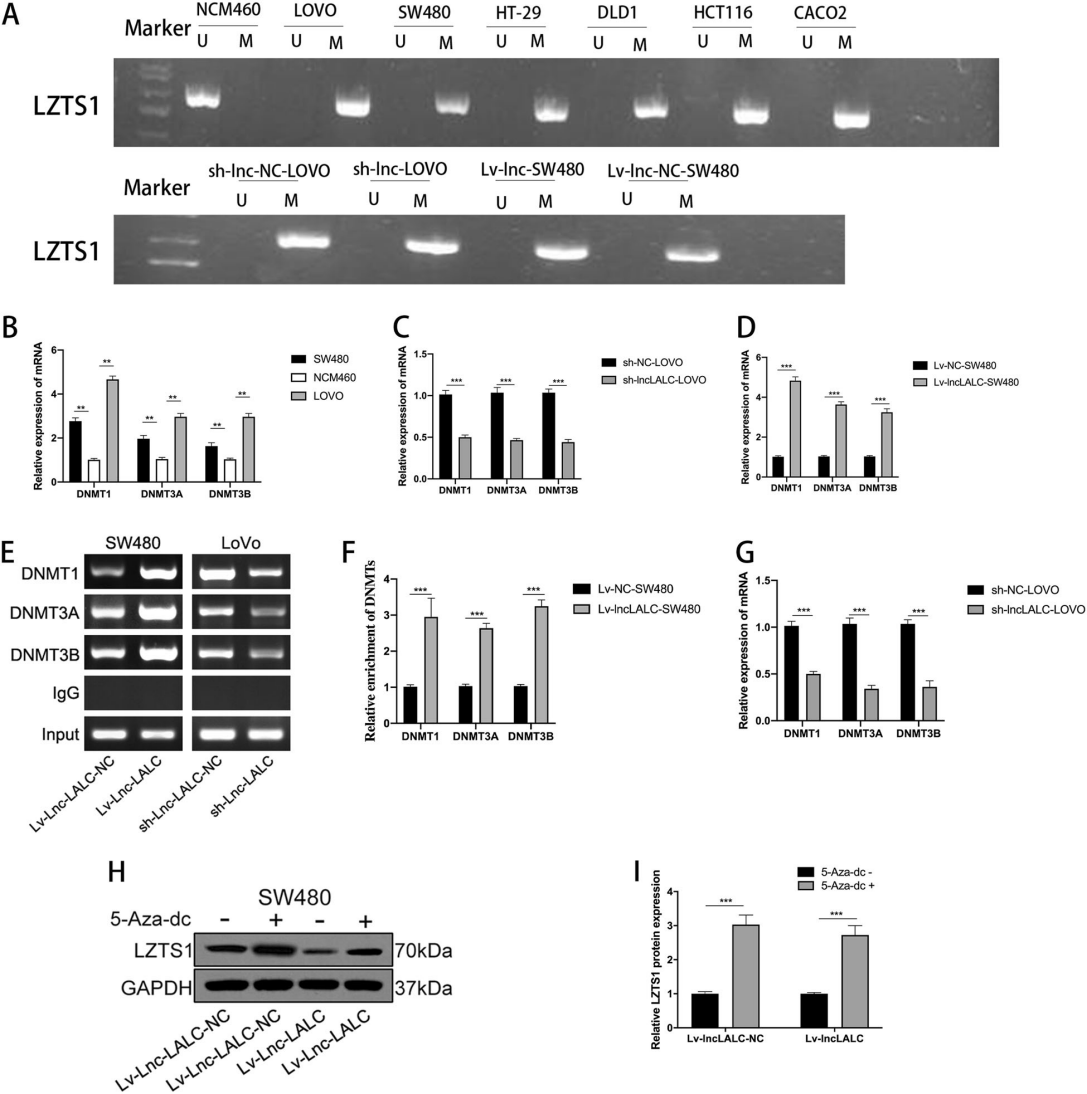
Lnc-LALC regulated methylation of LZTS1 promoters via binding with DNA methyltransferases. **A** Methylation specific PCR (MSP) revealed that the promoter region of LZTS1 was hypermethylated in CRC cells but hypomethylated in the NCM460 cells; and also revealed that the methylation level has a positive correlation with lnc-LALC expression level. U means unmethylated;M means methylated. **B** The expression level of DNMTs (DNMT1, DNMT3A, DNMT3B) was detected by qRT-PCR in SW480, LoVo cells, and NCM460 cells. **C** qRT-PCR assay showed knockdown of lncLALC could inhibit expression of DNMTs in LoVo cells. **D** qRT-PCR assay showed overexpression of lncLALC could promote the expression of DNMTs in SW480 cells. **E**–**G** ChIP assays revealed that the enrichment of DNMT1, DNMT3A, DNMT3B in LZTS1 promoter increased significantly after lncLALC overexpression in SW480 cells and reduced dramatically by lncLALC knockdown in LoVo cells. **H**, **I** Western blotting showed that methylation inhibition by 5-aza-2′-deoxycytidine (5-Aza-dC) could restore LncLALC-induced LZTS1 suppression. *P < 0.05; **P < 0.01; ***P < 0.001.

The methylation-specific PCR (MSP) revealed that the promoter region of *LZTS1* was hypermethylated in CRC cells, but hypomethylated in the NCM460 cells. We then used the same method to detect the methylation level in sh-*lncLALC*-LoVo, sh-NC-LoVo, Lv-*lncLALC*-SW480, and Lv-NC-SW480 cell lines. We found that there were more unmethylated CpG islands in the promoter of *LZTS1* in sh-*lncLALC*-LoVo cells than sh-NC-LOVO cells and more methylated CpG islands in Lv-*lncLALC*-SW480 cells than Lv-NC-SW480 cells (Fig. 5A). These results revealed that there was a positive correlation between *Lnc-LALC* expression and the methylation of *LZTS1* promoter and that DNA methylation may played important roles in the progression of *Lnc-LALC* epigenetically suppressing *LZTS1* expression.

The main process of DNA promoter methylation is a reversible process in which methyl groups on S-Adenosine methionine are attached to cytosine or adenine to form mC under the catalyzing of DNMTs(including DNMT1, DNMT3A, and DNMT3B). Thus, changes in DNMTs activity or expression may induce numerous diseases including CRC. To explore whether DNMTs participate into the regulation of *LZTS1* methylation regulated by *Lnc-LALC*, we firstly detected the expression levels of DNMTs and found that the levels were higher in SW480 and LoVo cells than NCM460 cells (Fig. 5B). The expression levels of DNMT1, DNMT3A, and DNMT3B were lower in sh-*lncLALC*-LoVo cells than that in sh-NC-LoVo cells and higher in Lv-NC-SW480 cells than that in Lv-*lncLALC*-SW480 cells (Fig. 5C, D). ChIP assays also revealed that the enrichment of DNMT1,DNMT3A,DNMT3B in *LZTS1* promoter increased significantly after *lnc-LALC* overexpression in Lv-lncLALC-SW480 cells and reduced dramatically after *lnc-LALC* knockdown in sh-lncLALC-LoVo cells (Fig. 5E). 5-aza-2′-deoxycytidine (5-Aza-dC), an effective inhibitor of DNMTs, was used to treat Lv-lncLALC-SW480 and Lv-NC-SW480 cells. The methylation inhibition could restore *LncLALC*-induced *LZTS1* suppression (Fig. 5F). These data suggested that *Lnc-LALC* was involved in the regulation of *LZTS1* methylation mediated by DNMTs and then changed the expression of *LZTS1*.

### *Lnc-LALC* recruited DNA Methyltransferases to the *LZTS1* promoter regions by combining with EZH2

**Fig. 6:**
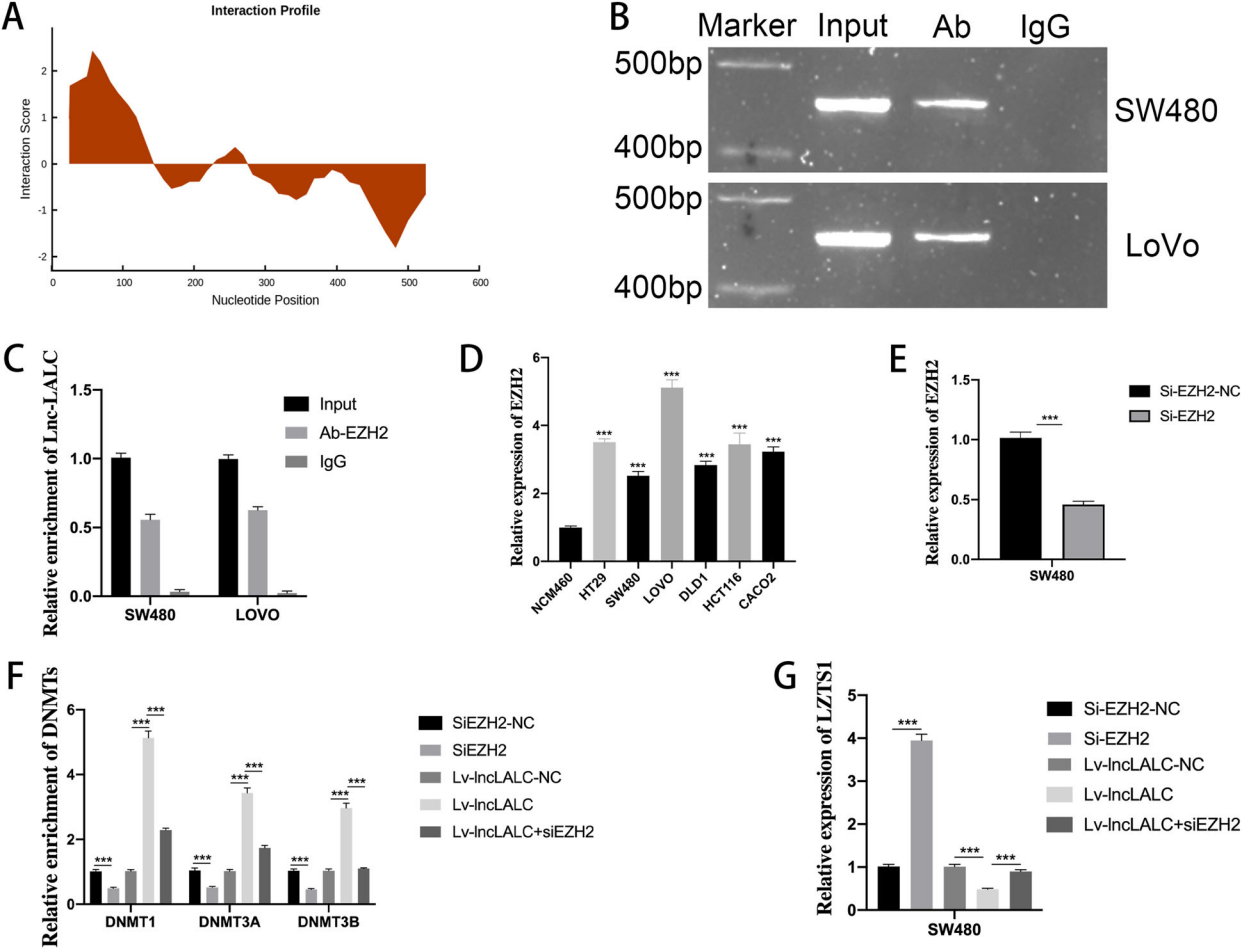
LncLALC recruited DNA methyltransferases to the LZTS1 promoter regions by combining with EZH2. **A** Preliminary prediction by bioinformatics system showed that Lnc-LALC may have certain binding ability to EZH2. (http://service.tartaglialab.com/page/catrapid_group). **B**, **C** RIP test using the EZH2 antibody showed that there was a strong combining capacity between lncLALC and EZH2 in SW480 cells and LOVO cells. **D** qRT-PCR showed that EZH2 was elevated in CRC cells than NCM460 cells. **E** The silencing efficacy of EZH2 in SW480 cells was determined by qRT-PCR. **F** The CHIP results showed that siEZH2 could reduce the enrichment of DNMT1, DNMT3A, DNMT3B in LZTS1 promoter and could also reverse the DNMTs^,^ high enrichment induced by Lv-lncLALC in SW480 cells. **G** qRT-PCR showed that siEZH2 could increase LZTS1 expression level and abolish the suppression of LZTS1 induced by Lv-lncLALC in SW480 cells. *P < 0.05; **P < 0.01; ***P < 0.001.

Numerous studies have reported that lncRNAs altered gene expression through interacting with polycomb repressive complex 2(PRC2) to promote malignant tumor invasiveness and metastasis^29^. Enhancer of zeste homolog 2 (EZH2), an important member of PRC2, is frequently reported to recruit long no coding RNAs in many tumors^30^. The preliminary prediction of the binding ability of *Lnc-LALC* to EZH2 by bioinformatics system showed that *Lnc-LALC* may have certain binding ability to EZH2 (Fig. 6A). We then performed RIP to test whether *lnc-LALC* could regulate *LZTS1* expression by recruiting EZH2 to its promoter and found that there is a strong combining capacity between *lnc-LALC* and EZH2 in SW480 cells and LOVO cells (Fig. 6B, C).

To further determine the mechanisms of EZH2 between *lnc-LALC* and *LZTS1*, we firstly tested EZH2 expression and found that EZH2 was elevated in CRC cells (Fig. 6D). We then used siEZH2 to silence EZH2 in SW480 cells and the silencing efficacy was determined by qRT-PCR (Fig. 6E). The CHIP results showed that siEZH2 not only reduced the enrichment of DNMT1, DNMT3A, DNMT3B in *LZTS1* promoter but also reversed the DNMTs, high enrichment induced by Lv-*lncLALC* in SW480 cells (Fig. 6F). Accompanied by these findings, siEZH2 could increase *LZTS1* expression level and abolish the suppression of *LZTS1* induced by Lv-*lncLALC* in SW480 cell lines (Fig. 6G). In all, *lnc-LALC* could recruit DNMTs to the *LZTS1* promoter by combining with EZH2 and then alter the expression of *LZTS1* via DNMTs-mediated DNA methylation.

### *Lnc-LALC* regulated epithelial–mesenchymal transition (EMT) in CRC cells through *LZTS1*

**Fig. 7:**
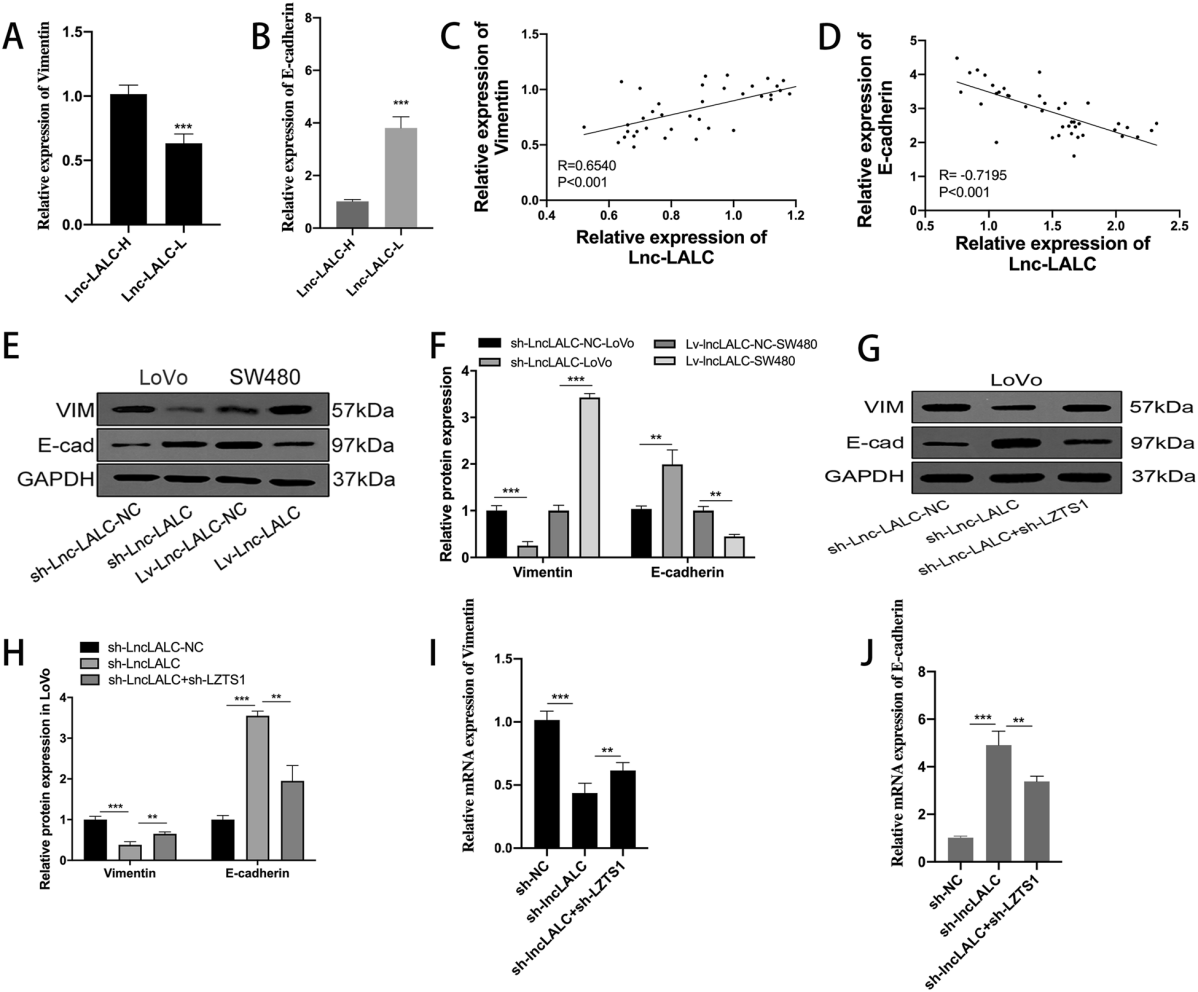
LncLALC regulated Epithelial–mesenchymal transition (EMT) in CRC cells through LZTS1. **A** The qRT-PCR results showed that Vimentin was overexpressed in lncLALC High group than lncLALC Low group. **B** The qRT-PCR results showed that E-cadherin was lower expressed in lncLALC High group than in LncLALC low group. **C** The Pearson correlation analysis confirmed that lncLALC expression had a positive correlation with Vimentin expression (*r* = 0.6540, *P* < 0.001). **D** The Pearson correlation analysis confirmed that lncLALC expression had a negative correlation with E-cadherin expression (*r* = −0.7195, *P* < 0.001). **E**, **F** Western blot assays showed that sh-LncLALC could enhanced the expression of E-cadherin but reduced the expression of Vimentin in LoVo cells and Lv-Lnc-LALC had the opposite effects in SW480 cells. **G**, **H** Western blot assays showed that knockdown of LZTS1 could restore the function of sh-lncLALC on EMT markers in LoVo cells. **I** qRT-PCR assays showed that knockdown of LZTS1 could restore the function of sh-lncLALC on Vimentin in LoVo cells. **J** qRT-PCR assays showed that knockdown of LZTS1 could restore the function of sh-lncLALC on E-cadherin in LoVo cells.

EMT plays an important role in the invasion and metastasis progress in various epithelial tumors, including CRC^31,32^. To verify whether *Lnc-LALC* can promote the invasion and metastasis of CRC by regulating the expression of *LZTS1*, we randomly selected 40 CRC tissues in two groups(20 samples in High group and 20 samples in Low group, the two groups were divided according to their average *Lnc-LALC* expression level in tumor tissues) and performed qRT-PCR to detect the expression level of Vimentin (a mesenchymal marker) and E-cadherin (an epithelial marker) in these two groups. qRT-PCR showed that Vimentin was overexpressed in *lnc-LALC* High group than *lnc-LALC* Low group and E-cadherin was lower expressed in *lnc-LALC* High group than in *Lnc-LALC* low group (Fig. 7A, B). *Lnc-LALC* was positively correlated with Vimentin expression (*r* = 0.6540, *P* < 0.001) and negatively correlated with E-cadherin expression (*r* = − 0.7195, *P* < 0.001) (Fig. 7C, D). Additionally, the following western blot assays showed that sh-*LncLALC* could enhance the expression of E-cadherin but reduced the expression of Vimentin in LoVo cells and Lv-*LncLALC* had the opposite effects in SW480 cells (Fig. 7E, F). To explore whether *LZTS1* could mediate the function of *Lnc-LALC* on EMT, we inhibited *LZTS1* expression in sh-LncLALC LoVo cells by sh-LZTS1. Western blot assays and qRT-PCR showed that knockdown of *LZTS1* could restore the function of sh-*lncLALC* on EMT markers in LoVo cells (Fig. 7G–J). Collectively, *Lnc-LALC* could promote EMT phenotype in CRC cells mediated by *LZTS1*.

## Discussion

CRC is one of the most common malignancies and its morbidity and mortality increased dramatically in the past decades. Distant metastasis is the leading cause of cancer-related death of CRC and liver is the primary metastasis organ compared with other organs. Van Cutsem reported that the survival of CRLM without treatment with a poor survival outcome as 5-12 moths^33^. Even though CRLM lesion could be respected, the recurrence rate and distant metastases are about 50% after operation^34^. Thus, it is essential to understand the deep mechanisms underlying CRLM to excavate more molecular targets and potential biomarkers for the diagnosis and therapy of CRLM.

*LZTS1* is one proved suppressor in CRC by inhibiting the initiation and proliferation of CRC cells. In the present study, we further investigated the aberrant expression of *LZTS1* in CRC tissues and cell lines and clarified more functions in the progression of CRLM. The preliminary experimental results showed that *LZTS1* was downregulated in CRLM tissues and CRC cells compared normal tissues and epithelial cell lines. These data suggested that strategies which can inhibit the downregulation of *LZTS1* may be effective measures to control CRLM development. Thus, more investigations should be done to reveal the underlying mechanisms of *LZTS1* in CRLM.

Numerous studies have indicated that lncRNAs could regulate the tumorigenesis and metastasis of a variety of cancers through modulating gene expression with epigenetic alteration^35,36^. Based on these theories, we hypothesized there might exist any lncRNAs which can modify the role of *LZTS1* in CRLM. To verify the assumption, we performed microarray analysis to explore the differentially expressed lncRNAs between the CRLM group and the CRC group. Through bioinformatics analysis, we found that there were 4 LncRNAs significantly correlated with the progression of CRC and may play a unique role in the liver metastasis of CRC. Furthermore, we found that the expression of *lnc-LALC* was negatively correlated with that of *LZTS1* through the detection of histological expression and Pearson correlation analysis. This suggested that it might play a unique role in the action of *LZTS1* and we named it *Lnc-LALC*. Firstly, we confirmed the non-coding ability by using bioinformatics software prediction. We then analyzed the association between *Lnc-LALC* expression and the clinical characteristics of CRC patients according to the average *Lnc-LALC* expression level in tumor tissues of 120 CRC patients and found that high expression of *Lnc-LALC* can promote nodal metastasis, vascular invasion, advanced stage, liver metastasis. The followed experiments indicated that *Lnc-LALC* could promote CRC cells migration and invasion in vitro and vitro. To sum up, *Lnc-LALC* plays critical roles in the metastasis of CRC.

DNA methylation is one intensely studied epigenetic modifications and can regulate gene expression and gene silencing properly^37^. As we all know, hypermethylation within the promoter regions can lead to the inactivation of some tumor-suppressor genes and this phenomenon occurs commonly in multiple cancers^37^. We have confirmed that *LZTS1* was a suppressor gene in CRLM progression and higher methylation levels of *LZTS1* in the CRLM group compared non-metastatic CRC group and controls. Followed experiments showed there was a significant correlation between *lnc-LALC* and *LZTS1* expression, so we hypothesized that *LZTS1* methylation may play important role in CRC metastasis and *lnc-LALC* may participate in this progress. Next, a series of assays demonstrated that *Lnc-LALC* epigenetically suppress *LZTS1* expression via promoting *LZTS1* methylation and through interacting with DNA methyltransferases.EZH2, a methyltransferase, and component of the PRC2, contributing to many essential biological processes of epigenetic maintenance and has been a candidate oncogene in various cancers^38,39^. We then determined the mechanisms of EZH2 between *lnc-LALC* and *LZTS1* and identified that *lnc-LALC* could recruit DNMTs to the *LZTS1* promoter by combining with EZH2 and then alter the expression of *LZTS1* via DNMTs-mediated DNA methylation. The following experimental results further indicated that *lnc-LALC* could EMT phenotype in CRC cells mediated by *LZTS1*.In all, we could get the conclusion that Long non-coding RNA *Lnc-LALC* facilitates CRC liver metastasis through EMT via epigenetically silencing of *LZTS1* via regulating methylation of *LZTS1* promoters mediated by DNMTs.

## Materials and methods

### Patients and tissue specimens

All clinical data, materials, and tissue samples used in this study were collected with the approval of the ethics committee of the first affiliated hospital of Nanjing medical university. 80 primary tumor samples without liver metastasis, 40 primary tumor samples with liver metastasis of CRC patients and 40 normal epithelial tissues of the large intestine of healthy persons were collected in our hospital between January 2013 and January 2015. Samples were frozen in liquid nitrogen within 5 min after resection. Screening criteria: (a) Age18–80; (b) Qualitative diagnosis: the postoperative pathological diagnosis of the primary lesion was adenocarcinoma(T1-T4a) in the CRLM group; The hepatic lesion was metastatic adenocarcinoma; (c) Preoperative evaluation staging of CT scan and colonoscopy: T stage of the primary lesion was T1-T4a in both groups; The hepatic lesion was resectable in CRLM group; There was no suspicious metastatic lesion in the liver and was confirmed by intraoperative ultrasound. (d) None of the participants had a history of cancer or had received radiotherapy, chemotherapy, radiofrequency ablation, or other treatments before surgery.

### Cell lines and cell culture conditions

The human colorectal carcinoma LoVo, CACO2, DLD1, HT29,HCT116 and SW480 cell lines and colon epithelial cell line NCM460 were purchased from the Cell Bank of Chinese Academy of Sciences (Shanghai). CRC cells and NCM460 cells were respectively incubated in DMEM medium (Hyclone, Logan, UT, USA) and in McCoy’s 5a medium containing 10% fetal bovine serum (FBS; Gibco, USA) and 50 U/ml penicillin and streptomycin at 37 °C with 5% CO_2_.

### Quantitative real-time PCR

Total RNA was isolated from tissues or cell cultures using TRIzol reagent (Invitrogen, USA). PrimeScript RT reagent (Takara, Japan) was used for synthesizing cDNA. Quantitative real-time PCR was performed on an Applied Biosystem 7500 Real-time PCR system (Applied Biosystems, Foster City, CA, USA) using SYBR-Green Master (Roche). The specific oligonucleotide primer sequences (Generay Biotechnology, Shanghai) are presented in Supplementary Table 3. Glyceraldehyde 3-phosphate dehydrogenase (GAPDH) was used as an internal control and the qRT-PCR result was calculated by the 2^–ΔΔCT^ method.

### Cell invasion and migration assays

We selected LoVo for its high Lnc-LALC expression and SW480 for its low Lnc-LALC expression to conduct Transwell assays. Cell invasion and migration assays were measured using transwell chambers (8.0 μm, 24-well format; Corning) coated with 50 μL of 1 mg/mL diluted Matrigel (BD Biosciences) or not as invasion assay or migration assay. Cells (4 × 104) suspended in 100 μL of serum-free medium were added in the upper chamber while 600 μL of medium with 10% fetal bovine serum were plated to the lower chamber. All the chambers cultured in 37 °C, 5% CO_2_ incubator for 24 h. The cells remaining in the upper chamber were removed with a cotton swab and the migrated or invaded cells were stained with 1% crystal violet for 20 min after fixed with methanol. A light microscope was used to count the number of cells on the membrane.

### Wound-healing assay

We selected LoVo for its high *Lnc-LALC* expression and SW480 for its low *Lnc-LALC* expression to conduct wound-healing assays. Cells were seeded in 6-well plates with a density of 1 × 10^6^/well and grown to 90% confluence. Wounded gaps over the adherent cells were formed by sterile pipette tips of 100 μL(Corning, USA). The wound gaps were washed by phosphate-buffered saline (PBS) photographed at 0 h and 48 h after culturing by an Olympus camera system and the migrating ratio was calculated by imagepro-plus 6.0 (migrating ratio = [Average width of the linear wound at 0 h-Average width of linear wound at 48 h]/Average width of linear wound at 0 h). All the assays were performed three times in triplicate.

### Methylation specific PCR (MSP)

For MSP procedures, 2 μg of genomic DNA of CRC cells were collected, and incubated with bisulfite DNA Lysis Buffer for 1 h at 37 °C. Then, the samples were denatured, and Bisulphite deaminated. And then specifically designed primers were used for PCR amplification (Supplementary Table 3). The resulting PCR fragments were visualized by agarose gel electrophoresis.

### Bisulfite sequencing PCR (BSP)

The methylation status of *LZTS1* promoter was determined by Bisulfite sequencing PCR (BSP).DNA was extracted and digested with EcoRV (Takara). EpiTect Bisulite Kit (Qiagen, CA, USA) was used to perform the bisulfite sequencing analysis with the EpiTect Bisulite Kit (Qiagen, CA, USA) by the provider’s manual. The transformed DNA was then PCR-amplified using the TaKaRa rTaq Kit (TaKaRa). Primers were showed in Supplementary Table 3. Products of amplified PCR were then purified and cloned into pMD19-T (TaKaRa, Dalian, China).

### Chromatin immunoprecipitation (CHIP) assay

Chromatin immunoprecipitation (CHIP) assay was performed using the ChIP kit (Millipore, USA) according to the manufacture’s protocol. Cells were collected and cross-linked with 1% formaldehyde for 10 min at 37 °C. After washed 3 times with protease inhibitors contained by PBS for 10 min. Then, we used CHIP incubation buffer to keep cells suspending and centrifuged the cellular lysates for 20 min. The precipitate was incubated with DNMT1, DNMT3A, DNMT3B antibodies with magnetic beads overnight at 4 °C and IgG was used as negative control. The precipitate DNA was extracted and subjected to PCR after washed three times.

### Hematoxylin and eosin staining

Murine tumor tissues were embedded in paraffin. Paraffin-embedded sections were stained with hematoxylin-eosin and cut into 4 μm slides. We used a microscope (Olympus Corporation, Tokyo, Japan) to perform the histological evaluation.

### Lentivirus production and cell transfection

The lnc-LALC shRNA and LZTS1 shRNA sequences were cloned into lentivirus vector GV248 (Genepharma manufacturer, Shanghai, China), respectively.The negative control shRNAs without sequences for lnc-LALC and LZTS1 were also designed by this company.Sequences of lncL-ALC and LZTS1 were subcloned into the lentiviral vector GV367 for the overexpression of lnc-LALC and LZTS1 by Genepharma manufacturer (Shanghai, China). The negative control lentivirus vector without sequences were also designed in this method. All vectors were labeled with luciferase. Target cells (1 × 105) were transfected with lentivirus/medium.ratio of 1:50 in the presence of 5ug/ml polybrene. Cells used for the recovery experiment were transfected with two lentiviruses at the same time. The transfection efficiency was determined by qRT-PCR.48 h later, puromycin (2 μg/mL) was used to construct stable clones for 2 weeks. Each experiment was conducted three times, and data were averaged.

### Western blotting

According to the manufacture’s protocols, total proteins were extracted from CRC cells and tissue using RIPA lysis buffer (Keygen Biotech). Different amounts of proteins were separated on sodium dodecyl sulfate-polyacrylamide gel electrophoresis (SDS-PAGE) based on their molecular weight and subsequently transferred to polyvinylidene fluoride membranes. Then, the membranes were blocked with 5% non-fat milk dissolving in Tris-buffered saline for 2 h at room temperature and incubated with specific primary antibodies at 4˚C overnight. The membranes were incubated in rabbit or mouse secondary antibodies at room temperature for 2 h after rinsed in TBST for three times. The protein bands on the membranes were visualized on Enhanced Chemiluminescence Plus (EMD Millipore, Billerica, MA, USA).) with a Bio-Imaging System. The primary antibodies were anti-LZTS1 (ab226335, 1:2000), anti-EZH2 (ab186006, 1:1000), anti-Vimentin (ab92547, 1:1000), anti-E-cadherin (ab40772, 1:5000), GAPDH as control (ab9485, 1:1000).

### RNA immunoprecipitation (RIP) assay

RIP was performed using a Magna RIP RNA-Binding Protein Immunoprecipitation Kit (Millipore, USA) according to the manufacture’s protocols. CRC cells were collected and lysed by the RIP lysis buffer, then incubated with the magnetic beds and anti-EZH2 antibodies overnight at 4 °C. After the precipitate was washed by RIP wash buffer for six times, co-immunoprecipitated RNA in the precipitate was extracted and performed qRT-PCR analysis. Immunoglobulin G RIP of cells served as the negative control.

### Animal studies

48 BALB/c nude mice (5 weeks old, male) were purchased from Animal Center of Nanjing medical university and were randomly divided into six groups (8 in each group) including Lv-NC-SW480,Lv-*lncLALC*-SW480, Lv-*lncLALC* + Lv-*LZTS1*-SW480 groups and sh-NC-LoVo, sh-*lncLAL*C-LoVo, sh-*lncLALC* + sh-*LZTS1*-LOVO groups. The different cell group used stable cell lines infected by different lentivirus respectively. The mice were anesthetized by 5% chloral hydrate (intraperitoneal) and Isoflurane (respiratory inhalation). We made a 5 mm incision in the left upper abdomen of the mice and then took out the lower pole of the spleen. Six different types of cells were suspended in 200 μL PBS contained about 2 × 10^7^ cells were injected into the subcapsule of the spleen and the abdominal cavity was closed after oppression hemostasis for 3 min. All the mice were fed in SPF environment with a free diet after the operation. The mice were killed 6 weeks after tumor injection and the livers were collected for H&E staining and tumor numbers count.

### Immunohistochemistry (IHC)

All the specimens were fixed by 4% formalin and embedded in paraffin and then made into 4 μm sections. All the samples were dewaxed by pure xylene for 10 min twice and then soaked by ethanol solution. The sections were washed by PBS for 5 min two times. Tissue samples were sealed with 3% H_2_O_2_ solution at room temperature for 10 min, and then washed with PBS solution at room temperature. Using sodium citrate buffer solution (PH = 6.0) to do antigen repair at 95 °C water bath for 15 min. Wash samples with PBS solution for 3 times after reheating,2 min each time. The samples were sealed with 5% BSA solution for 15 min at 37 °C. The sections were incubated with *LZTS1* primary antibodies (1:1000, Abcam, USA) at 4 °C overnight. After washing with PBS 3 times, the sections were incubated with HRP‐polymer‐conjugated secondary antibody(Biotinylated HRP IgG) at room temperature for 1 h and then washed with PBS. The sections were dyed with Diaminobenzidine (DAB) for 5 min. Hematoxylin re-dyed the sections for 20 s, followed by flushing with running water. Dehydrated with alcohol and finally treated with xylene transparently. Using neutral gum to seal the sections. Using Image-Pro Plus software to do semi-quantitatively analysis of the *LZTS1* dyeing strength. In this study, we selected the area of interest (AOI) to measure its integrated optical density (IOD). Every index was detected for three times.

### Datasets

CRC microarray data of lncRNAs of 435 patients were downloaded from The Cancer Genome Atlas (TCGA). Based on the corresponding clinical data and American Joint Committee on Cancer (AJCC) TNM Staging Classification for Colon and rectal Cancer 8th ed., 2017, we divided the data into three groups:TanyNanyM1 (Any T stage, Any N stage with Distant metastases), TanyN+M0 (Any T stage with regional lymph node metastasis, no distant metastasis),T_any_N0M0 (Any T stage, No regional lymph node metastasis, no distant metastasis).

### Statistics analysis

All statistical analyses were performed by SPSS 13.0 software (Chicago, IL, USA) and GraphPad Prism software (La Jolla, CA, USA) using a two-tailed Student’s *t* test or Pearson’s correlation. The global *p*-value was controlled in multiple comparisons using *t*-tests. Data from three independent experiments were presented as the mean ± SD. Differences were statistically significant at *P* < 0.05.

## Supplementary information

Supplementary Table 1

Supplementary Table 2

Supplementary Table 3
