# Supplementary material for: Long non-coding RNA Lnc-LALC facilitates colorectal cancer liver metastasis via epigenetically silencing LZTS1
Source: Cell Death Dis. 2021 Feb 26;12(2):224. doi: 10.1038/s41419-021-03461-w (PMC7910484; doi:10.1038/s41419-021-03461-w)
Supplement: Supplementary file 1 — Supplementary Table 1 [file 41419_2021_3461_MOESM1_ESM.pdf]

Supplementary Table 1. Aberrant lncRNAs identified by VENNY analysis

Gene	Location
LINC01101	chr2:120464335-120466349
LINC02418	chr12:130033812-130042342
LINC00922	chr16:65284499-65576300
MIR31HG	chr9:21454268-21559833
