# Supplementary material for: Long non-coding RNA Lnc-LALC facilitates colorectal cancer liver metastasis via epigenetically silencing LZTS1
Source: Cell Death Dis. 2021 Feb 26;12(2):224. doi: 10.1038/s41419-021-03461-w (PMC7910484; doi:10.1038/s41419-021-03461-w)
Supplement: Supplementary file 2 — Supplementary Table 2 [file 41419_2021_3461_MOESM2_ESM.pdf]

Supplementary Table 2.The relationship between Lnc-LALC expression and the clinicopathologic features of CRC samples

Characteristics	Lnc-LALC expression levels		P value
	Low(n=60)	High(n=60)	
Age			>0.05
⩽ 65	27	29	
>65	33	31	
Gender			>0.05
Male	37	32	
Female	23	28	
Tumor size(cm)			>0.05
⩽5	28	35	
>5	32	25	
T factor			<0.01
1 + 2	40	17	
3 + 4	20	43	
N factor			<0.01
0	41	10	
1+2	19	50	
M factor			<0.01
0	55	25	
1	5	35	
Vascular invasion			<0.05
Yes	22	36	
No	38	24	
Stage			<0.01
I + II	43	8	
III + IV	17	52	
CEA (ng/ml)			<0.01
< 4.7	36	14	
⩾ 4.7	24	46	

The median expression level of Lnc-LALC was used as the cutoff
