# Supplementary material for: Long non-coding RNA Lnc-LALC facilitates colorectal cancer liver metastasis via epigenetically silencing LZTS1
Source: Cell Death Dis. 2021 Feb 26;12(2):224. doi: 10.1038/s41419-021-03461-w (PMC7910484; doi:10.1038/s41419-021-03461-w)
Supplement: Supplementary file 3 — Supplementary Table 3 [file 41419_2021_3461_MOESM3_ESM.pdf]

Supplementary Table 3. Primer sequences used in this study

Gene		Primer sequence
LZTS1	Forward Primer	TGTCCAGCGGGGATTTAGG
	Reverse Primer	GCCCATTTCTAGCTGATTGGAGA
LINC00922	Forward Primer	CCATATCACCAGACCACAA
	Reverse Primer	TCACCATCACAGTAGAGAAC
DNMT1	Forward Primer	AGGCGGCTCAAAGATTTGGAA
	Reverse Primer	GCAGAAATTCGTGCAAGAGATTC
DNMT3A	Forward Primer	CCGATGCTGGGGACAAGAAT
	Reverse Primer	CCCGTCATCCACCAAGACAC
DNMT3B	Forward Primer	AGGGAAGACTCGATCCTCGTC
	Reverse Primer	GTGTGTAGCTTAGCAGACTGG
EZH2	Forward Primer	AATCAGAGTACATGCGACTGAGA
	Reverse Primer	GCTGTATCCTTCGCTGTTTCC
Vimentin	Forward Primer	AGTCCACTGAGTACCGGAGAC
	Reverse Primer	CATTTCACGCATCTGGCGTTC
E-cadherin	Forward Primer	CGAGAGCTACACGTTCACGG
	Reverse Primer	GGGTGTCGAGGGAAAAATAGG
LZTS1(MSP)	Methylated Left Primer	TGTTATGATTTTAGTTTTTTTACGG
	Methylated Right Primer	AAACTTACGCAACTTATACTACGAA
	Unmethylated Left Primer	TGTTATGATTTTAGTTTTTTTATGG
	Unmethylated right Primer	AAAAACTTACACAACTTATACTACAAA
LZTS1(BSP)	Methylated Left Primer	TTTAGTAGTTTTGGAAAAAGAGAGA
	Methylated Right Primer	TAAAATCATAACAAAACCCTCACAA
	Unmethylated Left Primer	TTTAGTAGTTTTGGAAAAAGAGAGA
	Unmethylated right Primer	ATAACAAAACCCTCACAAAACCTAC
